# Association Between Infant-Mother Room-Sharing and Symptoms of Postpartum Depression: A Population-Based Study

**DOI:** 10.1007/s10995-025-04073-y

**Published:** 2025-03-20

**Authors:** Sravya Patibandla, Zelalem T. Haile

**Affiliations:** 1 Ohio University Heritage College of Osteopathic Medicine, Dublin, OH USA; 2 Department of Social Medicine, Ohio University Heritage College of Osteopathic Medicine, 6775 Bobcat Way, Dublin, OH 43016 USA

**Keywords:** Room sharing, Infant sleeping arrangement, Postpartum depression, Maternal mental health

## Abstract

**Objectives:**

This study aimed to (1) examine the relationship between infant-mother room-sharing and postpartum depression (PPD) symptoms and (2) determine whether the relationship between infant-mother room-sharing and PPD symptoms varies by other maternal or infant characteristics.

**Methods:**

This cross-sectional study utilized de-identified secondary data from the 2016-2019 Pregnancy Risk Assessment and Monitoring System (PRAMS) (N=105,144). Frequencies and percentages were used to describe the characteristics of the study sample. Rao-Scott chi-square tests were used to examine differences in PPD symptoms and infant-mother room-sharing by maternal and infant characteristics. Multivariable logistic regression was performed to examine the independent association between infant sleeping arrangements and PPD symptoms. Pairwise interaction between infant sleeping arrangement and each covariate were included in the regression model, and stratified analyses were performed for variables with significant pairwise interactions.

**Results:**

The prevalence of PPD symptoms was 11.7%, and 79.5% reported that their infant sleeps in the same room. Significant pairwise interactions were found between infant-mother room-sharing and marital status, education, insurance, and receipt of WIC food assistance on PPD symptoms. The odds of having PPD symptoms were higher in those whose infants shared the same room compared to those whose infants slept in a different room. However, the observed association was present only in the subgroups of participants who were married, had greater than a high school level of education, had private insurance, and did not receive WIC food assistance during pregnancy.

**Conclusions for Practice:**

Findings suggest that infant-mother room-sharing is independently associated with increased odds of PPD symptoms.

## Significance

Infant-mother room-sharing has previously been correlated with poor sleep quality, and the literature reports a well-established relationship between poor sleep and depressive symptoms. Room-sharing between mother and infant has been proposed to impact maternal postpartum depression symptoms, but population-based research on this subject is lacking. After adjustment for maternal demographic, social, and health factors, as well as infant characteristics, mothers who room-shared with their infants were more likely to report postpartum depression symptoms. This relationship was limited to women of higher socioeconomic standing. This suggests infant-mother room-sharing may be associated with symptoms of postpartum depression.

## Introduction

Postpartum depression (PPD) is a common pregnancy-related complication affecting millions worldwide. It can disrupt a mother’s mental health and have long-lasting impacts on spousal relationships and child development (Faisal-Cury et al., 2021). Depression is on the rise in the United States (US) and has been for many years (Goodwin et al., 2022), with approximately 13% of mothers in the US reporting symptoms of PPD (Bauman et al., 2020). PPD has significant effects on both mother and child’s quality of life and is a significant risk factor for suicide (Agrawal et al., 2022). It is also a modifiable risk factor for various physical and mental diseases (Bica et al., 2017). Because of the widespread impact of PPD, a better understanding of its risk factors is imperative for the health and well-being of the public.

In 2016, the American Academy of Pediatrics (AAP) safe sleep guidelines were updated to recommend infants sleep in the same room as their parents for ideally one year, but at least six months, to reduce the number of sudden infant death syndrome (SIDS)-related deaths (AAP Task Force on Sudden Infant Death Syndrome, 2016). Recently, in 2022, the AAP again modified their guidelines to reduce the length of time parents should room-share with their infant from 1 year to 6 months (Moon et al., 2022). Room-sharing is supported by data showing that infants sleeping in the same room as their parents decrease the risk of SIDS by up to 50% (Moon & AAP Task Force on Sudden Infant Death Syndrome, 2016; AAP Task Force on Sudden Infant Death Syndrome, 2016). However, these guidelines may have unintended consequences for maternal mental health. Disrupted maternal sleep quality, which is associated with infant room-sharing, is a known risk factor for PPD (Leistikow & Smith, 2024). A few studies have shown an association between SIDS and PPD, furthering the importance of delineating if room-sharing is associated with PPD (Bandoli et al., 2022). Though there is data to support that infant-parent room-sharing decreases the risk of SIDS, the effect of room-sharing on the mother’s mental health has not been sufficiently explored. Previous studies have shown when infants sleep in the same room as their mother, the mother tends to have reduced sleep quality (Volkovich et al., 2015).

This study fills a gap in knowledge on the association between infant sleeping arrangements and PPD symptoms. Considering the long-lasting effects of PPD on both mother and infant (Suryawanshi & Pajai, 2022), evidence-based studies are needed to aid the development of strategies to reduce the incidence of PPD. This study aims to (1) examine the relationship between infant sleeping arrangements and symptoms of PPD in mothers and (2) determine whether the relationship between sleeping arrangement and symptoms of PPD varies by other sociodemographic, maternal health, maternal social, or infant health characteristics. We hypothesized that the presence of PPD symptoms differs by whether the infant sleeps in the same room as their mother. We further hypothesized that the relationship between infant sleeping arrangement and PPD symptoms in mothers would be moderated by maternal sociodemographic, maternal social, maternal health, and infant health characteristics.

## Methods

The data for this study was obtained from the Pregnancy Risk Assessment and Monitoring System (PRAMS), a population-based survey geared to collect data on both national and state-specific maternal and infant health issues and behavioral factors (*Pregnancy Risk Assessment Monitoring System* 2022). This study used PRAMS data from the years 2016–2019, during which the PRAMS survey was in its 8th phase. The survey was administered by 51 sites within the US and its surrounding territories, including data from 47 states (Shulman et al., 2018). At each site, between approximately 1000 to 3000 postpartum women were selected, contacted primarily by mail surveys, and then followed up with by telephone if there was no initial response (Shulman et al., 2018). The PRAMS survey is administered to new mothers between two and six months postpartum (Shulman et al., 2018). The survey and associated birth certificate data were deidentified and made available to researchers upon request. This study was classified as exempt by the Institutional Review Board of the author’s institution.

During 2016–2019, 164,403 women participated in the survey. For this study, only women whose infant was living at the time of the report were included in the study population. Participants who had more than one birth (i.e., twins, triplets) were omitted from the study population. Women who had missing responses for infant sleeping location, symptoms of PPD, or any covariates were also omitted from the study population. The final study population was 105,144 participants.

### Outcome of Interest

The dependent variable for this study was symptoms of PPD. To classify a participant as having symptoms of maternal PPD, the PPD indicator variable in PRAMS was used. The indicator considered responses to two questions, asking: “Since your new baby was born, how often have you felt down, depressed, or hopeless?” and “Since your new baby was born, how often have you had little interest or little pleasure in doing things you usually enjoyed?”. Participants had answer options of *Always*,* Often*,* Sometimes*,* Rarely*, or *Never*. Those who answered *Always* or *Often* to either question were coded as having PPD symptoms. These questions were derived from Patient Health Questionnaire 2 (PHQ-2), a shorter version of the PHQ-9, which provides acceptable accuracy for postpartum depression screening (Gigantesco et al., 2022). The PRAMS dataset uses slightly different answer options, offering five choices instead of four. Although these answer choices are not from an established PPD questionnaire, they have been used by the CDC PRAMS for many years to assess PPD symptoms.

### Independent Variable

The independent variable for this study was infant-mother room-sharing. Mothers were asked, “When your new baby sleeps alone, is his or her crib or bed in the same room where you sleep?”. Those who answered “yes” were coded as room-sharing, while those who responded “no” were coded as not room-sharing with their infant.

### Covariates

Covariates were selected based on the review of existing literature that include maternal and infant characteristics (Ay, 2018; Clout & Brown, 2015; Hutchens & Kearney, 2020).

*Maternal sociodemographic characteristics*: The following maternal sociodemographic characteristics were used as covariates: maternal age (< 20, 20–24, 25–34, 35+), marital status (married, not-married), maternal race/ethnicity (non-Hispanic white, non-Hispanic Black, Hispanic, other), maternal education (less than high school, high school, greater than high school), maternal postnatal insurance (none, Medicaid, private), and received Special Supplemental Nutrition Program for Women, Infants, and Children (WIC) food assistance during pregnancy (no, yes).

*Maternal Behavior*: Maternal behavior considered included pregnancy intention (intended, mistimed, unwanted, unsure), maternal postnatal checkup (no, yes), maternal postnatal smoking (no, yes), prepregnancy alcohol use (none, low-risk, heavy use), abused during pregnancy by husband/partner (no, yes), and mother ever breastfed (no, yes).

*Maternal Health Characteristics*: The following maternal health factors were used as covariates: parity (primiparous, multiparous), prepregnancy body mass index (BMI) (underweight, normal, overweight, obese), gestational weight gain based on IOM guideline (below guideline, within guideline, above guideline), Kotelchuck adequacy of prenatal care (inadequate, intermediate, adequate, adequate plus), mode of delivery (cesarean, vaginal), hypertension during pregnancy (no, yes), prepregnancy diabetes (no, yes), and gestational diabetes mellitus (GDM) (no, yes).

*Infant Characteristics*: The following infant health factors were used as covariates: infant gestational age (full-term, preterm), infant birth weight (low, average, high), and infant sex (male, female).

### Statistical Analysis

Frequencies and percentages were used to describe the characteristics of the study sample. Rao-Scott chi-square tests were performed to examine differences in PPD symptoms and infant sleeping arrangement by maternal and infant characteristics. Multivariable logistic regression analyses were fitted to examine the independent association between infant sleeping arrangement and PPD symptoms. Covariates with a p value < 0.05 in the bivariate analysis were retained in the multivariable regression. To examine differences in the association between sleeping arrangement and PPD symptoms by maternal and infant characteristics, pairwise interaction between infant sleeping arrangement and each covariate were included in the regression model. A stratified analysis was performed for variables with a significant pairwise interaction at *p* < 0.05. All analyses were adjusted for complex sample design elements of PRAMS, including stratification and weighting. All analyses were performed using SAS 9.4 (SAS Institute Inc, Cary, NC, USA).

## Results

The study sample consisted of 105,144 women. A notable proportion of the women indicated PPD symptoms (11.7%), while most women reported that their infant sleeps in the same room (79.5%). More than a half of women in the study sample were in the 25–34 age group (60.1%), were non-Hispanic white (62.9%), were married (64.9%), had greater than a high school education (67.9%), were not receiving WIC food assistance (67.5%), had private insurance (63.0%), and had an intentional pregnancy (60.6%). A description of the study sample is available in Table 1.

**Table 1:** Descriptive statistics of the study sample (*N* = 105144)

**Mother's age**	n (Wt.%)
< 20	4149 (3.7)
20–24	18965 (17.9)
25–34	62824 (60.1)
35 +	19206 (18.3)
**Marital status**	
Married	65516 (64.9)
Not married	39628 (35.1)
**Mother's race/ethnicity**	
Non-Hispanic White	54836 (62.9)
Non-Hispanic Black	17957 (13.5)
Hispanic	16848 (15.2)
Other	15503 (8.4)
**Parity**	
Primiparous	41498 (38.9)
Multiparous	63646 (61.1)
**Maternal education**	
Less than high school	10314 (9.1)
High school	23989 (23.0)
Greater than high school	70841 (67.9)
**Postnatal care insurance**	
None	9600 (10.3)
Medicaid	32612 (26.7)
Private	62932 (63.0)
**WIC food assistance during pregnancy**	
No	67496 (67.5)
Yes	37648 (32.5)
**Prepregnancy body mass index**	
Underweight	3627 (3.2)
Normal	46844 (45.7)
Overweight	26814 (25.7)
Obese	27859 (25.4)
**Gestational weight gain based on IOM guidelines**	
Below guideline	27566 (23.8)
Within guideline	31965 (30.7)
Above guideline	45613 (45.5)
**Kotelchuck adequacy of prenatal care**	
Inadequate	11700 (10.8)
Intermediate	10447 (10.2)
Adequate	46422 (46.5)
Adequate Plus	36575 (32.5)
**Pregnancy intention**	
Intended	62140 (60.6)
Mistimed	19763 (18.8)
Unwanted	6578 (6.0)
Unsure	16663 (14.6)
**Mode of delivery**	
Cesarean	35716 (32.2)
Vaginal	69428 (67.8)
**Maternal postpartum checkup**	
No	9437 (8.1)
Yes	95707 (92.0)
**Postpartum smoking**	
No	92991 (89.5)
Yes	12153 (10.5)
**Hypertension during pregnancy**	
No	93389 (90.8)
Yes	11755 (9.2)
**Prepregnancy diabetes**	
No	101642 (96.8)
Yes	3502 (3.2)
**Gestational diabetes mellitus**	
No	97822 (93.5)
Yes	7322 (6.5)
**Alcohol use before pregnancy**	
None	43706 (38.9)
Low-risk use	58603 (58.3)
Heavy use	2835 (2.8)
**Abused during pregnancy by husband/partner**	
No	102943 (98.2)
Yes	2201 (1.8)
**Mother ever breastfed**	
Yes	92872 (88.1)
No	12272 (11.9)
**Infant gestational age**	
Full term	89924 (92.6)
Preterm	15220 (7.4)
**Infant birth weight**	
Low	18247 (5.8)
Average	79203 (85.6)
High	7694 (8.6)
**Infant sex**	
Male	52995 (51.0)
Female	52149 (49.0)
**Infant-mother room-sharing**	
No	18901 (20.5)
Yes	86243 (79.5)
**Symptoms of postpartum depression**	
No	91612 (88.3)
Yes	13532 (11.7)

Abbreviations: Wt.%: Weighted percent; WIC: Special Supplemental Nutrition Program for Women, Infants, and Children; IOM: Institute of Medicine

Characteristics of the study sample by infant-mother room-sharing status are available in Table 2. Women who were younger, unmarried, had less than a high school education, received WIC food assistance during pregnancy, were Hispanic or non-Hispanic Black, or had Medicaid or no insurance were more likely to room-share than other groups. Table 3 contains the characteristics of the study sample by PPD symptoms. PPD symptoms were higher among women who were younger, unmarried, had high school or less than high school education, recipients of WIC food assistance during pregnancy, were non-Hispanic Black, and had Medicaid or no postnatal care insurance. The bivariate analysis shows that women who room shared were more likely to have PPD symptoms than women who did not (12.4% versus 9.1%; *p* < 0.001). Except for parity, hypertension during pregnancy, GDM, and infant sex, all variables were significantly associated with PPD symptoms (Table 3).

**Table 2:** Characteristics of the study sample by infant room-sharing (*N* = 105144)

	Infant-mother room-sharing		
	No n (Wt. %)	Yes n (Wt. %)	p
**Mother's age**			< 0.001
< 20	383 (9.9)	3766 (90.1)	
20–24	2402 (14.19)	16563 (85.8)	
25–34	12411 (22.5)	50413 (77.5)	
35 +	3705 (22.3)	15501 (77.7)	
**Marital status**			< 0.001
Married	14994 (25.6)	50522 (74.7)	
Not married	3907 (11.1)	35721 (88.9)	
**Mother's race/ethnicity**			< 0.001
Non-Hispanic White	13937 (26.9)	40899 (73.1)	
Non-Hispanic Black	1635 (9.0)	16322 (91.0)	
Hispanic	1490 (8.9)	15358 (91.1)	
Other	1839 (12.5)	13662 (87.5)	
**Parity**			< 0.001
Primiparous	8947 (24.2)	32551 (75.8)	
Multiparous	9954 (18.2)	53692 (81.8)	
**Maternal education**			< 0.001
Less than high school	550 (6.1)	9764 (93.9)	
High school	2373 (11.6)	21616 (88.4)	
Greater than high school	15978 (25.5)	54863 (74.5)	
**Postnatal care insurance**			< 0.001
None	794 (8.1)	8806 (91.9)	
Medicaid	2617 (8.9)	29995 (91.1)	
Private	15490 (27.5)	47442 (72.5)	
**WIC food assistance during pregnancy**			< 0.001
No	15667 (25.9)	51829 (74.1)	
Yes	3234 (9.4)	34414 (90.6)	
**Prepregnancy body mass index**			< 0.001
Underweight	521 (16.3)	3106 (83.7)	
Normal	9579 (23.2)	37265 (76.8)	
Overweight	4706 (19.8)	22108 (80.2)	
Obese	4095 (16.9)	23764 (83.1)	
**Gestational weight gain based on IOM guidelines**			< 0.001
Below guideline	4135 (17.2)	23431 (82.8)	
Within guideline	6161 (21.9)	25804 (78.1)	
Above guideline	8605 (21.3)	37008 (78.7)	
**Kotelchuck adequacy of prenatal care**			< 0.001
Inadequate	1341 (12.5)	10359 (87.5)	
Intermediate	1751 (19.5)	8696 (80.5)	
Adequate	9359 (22.6)	37063 (77.4)	
Adequate Plus	6450 (20.6)	30125 (79.4)	
**Pregnancy intention**			< 0.001
Intended	13313 (24.3)	48827 (75.7)	
Mistimed	2757 (15.7)	17006 (84.3)	
Unwanted	761 (12.6)	5817 (87.4)	
Unsure	2070 (14.4)	14593 (85.6)	
**Mode of delivery**			0.267
Cesarean	6103 (20.2)	29613 (79.8)	
Vaginal	12798 (20.7)	56630 (79.3)	
**Maternal postpartum checkup**			< 0.001
No	924 (11.5)	8613 (88.5)	
Yes	17977 (21.3)	77730 (78.7)	
**Postpartum smoking**			< 0.001
No	17440 (21.3)	75551 (78.7)	
Yes	1461 (13.9)	10692 (86.1)	
**Hypertension during pregnancy**			0.023
No	16996 (20.7)	76393 (79.3)	
Yes	1905 (19.2)	9850 (80.8)	
**Prepregnancy diabetes**			0.012
No	18389 (20.6)	83253 (79.4)	
Yes	512 (17.9)	2990 (82.1)	
**Gestational diabetes mellitus**			0.001
No	17766 (20.7)	80056 (79.3)	
Yes	1135 (18.2)	6187 (81.8)	
**Alcohol use before pregnancy**			< 0.001
None	5084 (13.0)	38622 (87.0)	
Low-risk use	13093 (25.1)	45510 (74.9)	
Heavy use	724 (29.8)	2111 (70.2)	
**Abused during pregnancy by husband/partner**			< 0.001
No	18694 (20.7)	84249 (79.3)	
Yes	207 (10.9)	1994 (89.1)	
**Mother ever breastfed**			< 0.001
Yes	17192 (21.0)	75680 (79.0)	
No	1709 (16.8)	10563 (83.2)	
**Infant gestational age**			< 0.001
Full term	16885 (21.0)	73039 (79.0)	
Preterm	2016 (14.8)	13204 (85.2)	
**Infant birth weight**			< 0.001
Low	2447 (12.8)	15800 (87.2)	
Average	14742 (20.6)	64461 (79.4)	
High	1712 (25.3)	5982 (74.7)	
**Infant sex**			0.162
Male	9681 (20.8)	43314 (79.2)	
Female	9220 (20.2)	42929 (79.8)	
**Symptoms of postpartum depression**			< 0.001
No	17005 (21.1)	74607 (78.9)	
Yes	1896 (15.9)	11636 (84.1)	

Abbreviations: Wt.%: Weighted percent

**Table 3:** Characteristics of the study sample by postpartum depression (*N* = 105144)

	Postpartum depression				
	No n (Wt. %)	Yes n (Wt. %)	p	Unadjusted OR (95% CI)	p
**Mother's age**			< 0.001		
< 20	3215 (78.8)	934 (21.2)		2.71 (2.35, 3.14)	< 0.001
20–24	15684 (83.2)	3281 (16.8)		2.03 (1.85, 2.24)	< 0.001
25–34	55463 (89.6)	7361 (10.4)		1.18 (1.08, 1.28)	< 0.001
35 +	17250 (91.0)	1956 (9.0)		Reference	
**Marital status**			< 0.001		
Married	58664 (90.5)	6852 (9.5)		Reference	
Not married	32948 (84.2)	6680 (15.8)		1.78 (1.68, 1.89)	< 0.001
**Mother's race/ethnicity**			< 0.001		
Non-Hispanic White	48840 (89.6)	5996 (10.4)		Reference	
Non-Hispanic Black	14880 (84.1)	3077 (15.9)		1.62 (1.50, 1.76)	< 0.001
Hispanic	14880 (88.8)	1968 (11.2)		1.09 (0.99, 1.19)	0.070
Other	13012 (84.2)	2491 (15.8)		1.61 (1.48, 1.76)	< 0.001
**Parity**			0.154		
Primiparous	36352 (88.6)	5146 (11.4)		Reference	
Multiparous	55260 (88.1)	8386 (11.9)		1.05 (0.98, 1.11)	0.151
**Maternal education**			< 0.001		
Less than high school	8521 (84.0)	1793 (16.0)		1.73 (1.57, 1.90)	< 0.001
High school	20008 (84.7)	3981 (15.3)		1.64 (1.53, 1.75)	< 0.001
Greater than high school	63083 (90.1)	7758 (9.9)		Reference	
**Postnatal care insurance**			< 0.001		
None	8261 (87.0)	1339 (13.0)		1.42 (1.28, 1.58)	< 0.001
Medicaid	26927 (83.7)	5685 (16.3)		1.84 (1.73, 1.96)	< 0.001
Private	56424 (90.4)	6508 (9.6)		Reference	
**WIC food assistance during pregnancy**			< 0.001		
No	60427 (90.5)	7069 (9.5)		Reference	
Yes	31185 (83.8)	6463 (16.2)		1.84 (1.73, 1.95)	< 0.001
**Prepregnancy body mass index**			< 0.001		
Underweight	3083 (86.4)	544 (13.6)		Reference	
Normal	41346 (89.4)	5498 (10.6)		0.75 (0.64, 0.88)	< 0.001
Overweight	23451 (88.2)	3363 (11.8)		0.85 (0.72, 0.99)	0.042
Obese	23732 (86.5)	4127 (13.5)		0.99 (0.84, 1.16)	0.903
**Gestational weight gain based on IOM guidelines**			0.001		
Below guideline	23708 (87.5)	3858 (12.5)		Reference	
Within guideline	28150 (89.1)	3815 (10.9)		0.86 (0.79, 0.93)	< 0.001
Above guideline	39754 (88.1)	5859 (11.9)		0.94 (0.88, 1.01)	0.098
**Kotelchuck adequacy of prenatal care**			< 0.001		
Inadequate	9828 (85.7)	1872 (14.3)		1.29 (1.17, 1.42)	< 0.001
Intermediate	9029 (87.5)	1418 (12.5)		1.10 (0.99, 1.22)	0.087
Adequate	40964 (88.9)	5458 (11.1)		0.96 (0.90, 1.03)	0.230
Adequate plus	31791 (88.5)	4784 (11.5)		Reference	
**Pregnancy intention**			< 0.001		
Intended	55860 (90.7)	6280 (9.3)		Reference	
Mistimed	16650 (85.6)	3113 (14.4)		1.65 (1.53, 1.78)	< 0.001
Unwanted	5185 (81.1)	1393 (18.9)		2.28 (2.05, 2.54)	< 0.001
Unsure	13917 (84.5)	2746 (15.5)		1.80 (1.66, 1.94)	< 0.001
**Mode of delivery**			0.002		
C-section	30839 (87.6)	4877 (12.4)		1.10 (1.04, 1.17)	0.002
Vaginal	60773 (88.6)	8655 (11.4)		Reference	
**Maternal postpartum checkup**			< 0.001		
No	7650 (82.1)	1787 (17.9)		1.74 (1.58, 1.91)	< 0.001
Yes	83962 (88.8)	11745 (11.2)		Reference	
**Postpartum smoking**			< 0.001		
No	81956 (89.1)	11035 (10.9)		Reference	
Yes	9656 (81.4)	2497 (18.6)		1.87 (1.73, 2.03)	< 0.001
**Hypertension during pregnancy**			0.062		
No	81466 (88.4)	11923 (11.6)		Reference	
Yes	10146 (87.4)	1609 (12.6)		1.10 (1.00, 1.21)	0.060
**Prepregnancy diabetes**			0.034		
No	88622 (88.3)	13020 (11.7)		Reference	
Yes	2990 (86.5)	512 (13.5)		1.18 (1.01, 1.37)	0.033
**Gestational diabetes mellitus**			0.227		
No	85256 (88.3)	12566 (11.7)		Reference	
Yes	6356 (87.6)	966 (12.4)		1.07 (0.96, 1.20)	0.223
**Alcohol use before pregnancy**			< 0.001		
None	37425 (86.8)	6281 (13.2)		Reference	
Low-risk use	51833 (89.5)	6770 (10.5)		0.77 (0.73, 0.82)	< 0.001
Heavy use	2354 (84.0)	481 (16.0)		1.25 (1.06, 1.48)	0.007
**Abused during pregnancy by husband/partner**			< 0.001		
No	90121 (88.7)	12822 (11.3)		Reference	
Yes	1491 (65.3)	710 (34.7)		4.17 (3.57, 4.87)	< 0.001
**Mother ever breastfed**			< 0.001		
Yes	81368 (88.6)	11504 (11.4)		Reference	
No	10244 (85.6)	2028 (14.4)		1.32 (1.21, 1.43)	< 0.001
**Infant gestational age**			< 0.001		
Full term	78715 (88.5)	11209 (11.5)		Reference	
Preterm	12897 (85.9)	2323 (14.1)		1.26 (1.16, 1.37)	< 0.001
**Infant birth weight**			< 0.001		
Low	15409 (84.6)	2838 (15.4)		Reference	
Average	69373 (88.5)	9830 (11.5)		0.72 (0.67, 0.78)	< 0.001
High	6830 (89.1)	864 (10.9)		0.68 (0.59, 0.77)	< 0.001
**Infant sex**			0.345		
Male	46160 (88.1)	6835 (11.9)		Reference	
Female	45452 (88.4)	6697 (11.6)		0.97 (0.92, 1.03)	0.341
**Infant-mother room-sharing**			< 0.001		
No	17005 (90.9)	1896 (9.1)		Reference	
Yes	74607 (87.6)	11636 (12.4)		1.42 (1.32, 1.54)	< 0.001

Abbreviations: OR: Odds Ratio; CI: Confidence Interval

In the multivariable-adjusted model, we found significant pairwise interaction between infant sleeping arrangement and marital status (p for interaction < 0.001), education (p for interaction = 0.047), postnatal insurance (p for interaction = 0.001), and receipt of WIC food assistance on postnatal depression (p for interaction = 0.010) on PPD symptoms. Table 4 presents the association between infant sleeping arrangement and PPD symptoms stratified by marital status, education, postnatal insurance, and receipt of WIC food assistance during pregnancy. The association between infant sleeping arrangement and PPD symptoms was significant only in the subgroups of women who were married, had more than high school education, had postnatal care insurance, and had not received WIC food assistance. Among women who were married, the odds of having PPD symptoms were higher in those who room shared compared to those whose infants slept in a different room OR (95% CI): 1.15 (1.04–1.27); *p* = 0.007). Among women who had greater than high school education, the odds of having PPD symptoms were higher in those who room shared compared to those whose infants slept in a different room 1.11 (1.01–1.22); *p* = 0.032). Among women who had insurance, the odds of having PPD symptoms were higher in those who room shared compared to those whose infants slept in a different room 1.13 (1.03–1.25); *p* < 0.013). Among women who did not receive WIC food assistance during pregnancy, the odds of having PPD symptoms were higher in those who room shared compared to those whose infants slept in a different room, 1.11 (1.00-1.22); *p* = 0.041). For all other subgroups we were not able to detect a significant association between infant sleeping arrangement and the presence of PPD symptoms.

**Table 4:** Association between infant-mother room-sharing and postpartum depression symptoms stratified by marital status, maternal education, postnatal insurance, and WIC food assistance during pregnancy (*N* = 105144)

Stratum		Postpartum Depression Symptoms		
		Adjusted OR (95% CI)	p	p for interaction
**Marital status** ^**b**^				< 0.001
Married (n = 65516)		1.15 (1.04, 1.27)	0.007	
Not married (n = 39628)		0.93 (0.80, 1.07)	0.295	
**Maternal education** ^**c**^				0.047
Less than high school (n = 10314)		1.07 (0.73, 1.57)	0.730	
High school (n = 23989)		0.96 (0.80, 1.15)	0.653	
Greater than high school (n = 70841)		1.11 (1.01, 1.22)	0.032	
**Postnatal care insurance** ^**d**^				0.001
None (n = 9600)		0.80 (0.57, 1.11)	0.180	
Medicaid (n = 32612)		0.96 (0.81, 1.14)	0.656	
Private (n = 62932)		1.13 (1.03, 1.25)	0.013	
**WIC food assistance during pregnancy** ^**e**^				0.010
No (n = 67496)		1.11 (1.00, 1.22)	0.041	
Yes (n = 37648)		0.98 (0.85, 1.15)	0.840	

Abbreviations: OR: Odds Ratio; CI: Confidence Interval

^a^ Reference group: No infant-mother room-haring

^b^ Model adjusted for mother’s age, maternal race/ethnicity, maternal education, postnatal care insurance, WIC food assistance during pregnancy, prepregnancy BMI, gestational weight gain based on IOM guidelines, Kotelchuck adequacy of prenatal care, pregnancy intention, mode of delivery, infant gestational age, infant birth weight, maternal postnatal checkup, postnatal smoking, prepregnancy diabetes, prepregnancy alcohol use, abused during pregnancy by husband/partner, and ever breastfed

^c^ Model adjusted for mother’s age, marital status, maternal race/ethnicity, postnatal care insurance, WIC food assistance during pregnancy, prepregnancy BMI, gestational weight gain based on IOM guidelines, Kotelchuck adequacy of prenatal care, pregnancy intention, mode of delivery, infant gestational age, infant birth weight, maternal postnatal checkup, postnatal smoking, prepregnancy diabetes, prepregnancy alcohol use, abused during pregnancy by husband/partner, and ever breastfed

^d^ Model adjusted for mother’s age, marital status, maternal race/ethnicity, maternal education, WIC food assistance during pregnancy, prepregnancy BMI, gestational weight gain based on IOM guidelines, Kotelchuck adequacy of prenatal care, pregnancy intention, mode of delivery, infant gestational age, infant birth weight, maternal postnatal checkup, postnatal smoking, prepregnancy diabetes, prepregnancy alcohol use, abused during pregnancy by husband/partner, and ever breastfed

^e^ Model adjusted for mother’s age, marital status, maternal race/ethnicity, maternal education, postnatal care insurance, prepregnancy BMI, gestational weight gain based on IOM guidelines, Kotelchuck adequacy of prenatal care, pregnancy intention, mode of delivery, infant gestational age, infant birth weight, maternal postnatal checkup, postnatal smoking, prepregnancy diabetes, prepregnancy alcohol use, abused during pregnancy by husband/partner, and ever breastfed

## Discussion

This study aimed to examine the association between infant-mother room-sharing and symptoms of maternal PPD. Infant-mother room-sharing was positively associated with PPD symptoms only in women who were married, had higher than a high school level of education, had private insurance, or did not receive WIC food assistance during pregnancy. The impact of room-sharing on PPD symptoms is likely modest for the overall population, as women who room-shared in the study had between a 1.11 to 1.15 higher odds of reporting PPD symptoms based on the analysis of the overall sample. The observed association is unsurprising, as infant-mother room-sharing can contribute to poorer sleep quality and duration for mothers (Volkovich et al., 2015), and mothers with worse sleep quality tend to have greater rates of PPD (Baattaiah et al., 2023; Okun & Lac, 2023). Based on these findings, it is plausible that room-sharing can influence PPD symptoms due to its effect on maternal sleep.

While sleep quality may explain these findings, the subgroup of women for which these results are relevant is puzzling, as PPD is more prevalent among women of lower socioeconomic status (SES) (Agrawal et al., 2022). The observed association between room-sharing and PPD symptoms in women who were married, had greater than high school education, had private insurance, and had no WIC food assistance during pregnancy implies this association is present only for women of higher SES. A systematic review of the relationship between postpartum sleep disturbance and PPD found a strong relationship between sleep disturbance and PPD in a predominantly “White, married or partnered, middle class, and/or socially advantaged” sample, showing sleep disturbance was significantly associated with PPD (Bhati & Richards, 2015). With most research on sleep quality limited to women of higher SES, the association between sleep quality and duration and PPD may not extend to all groups.

Poor sleep quality is likely associated with an increased risk of developing PPD, but findings vary by measurements for sleep quality and duration. When both maternal perceptions of sleep quality and objective sleep measures were compared against PPD symptoms, only women with subjective deficiencies had a relationship between sleep quality and PPD (Bei et al., 2010). In this study, approximately 90% of the study population was also “married or in a stable relationship” (Bei et al., 2010), with larger studies reporting similar results (Dørheim et al., 2009; Stremler et al., 2020). Subjective perceptions of sleep quality and duration over objective sleep disturbances may contribute to the association between room-sharing and PPD symptoms. Yet it should be noted that multiple studies on sleep quality and SES report pregnant and postpartum women in lower SES have less sleep and worse perceived sleep than women of higher SES (Okun et al., 2014).

The link between higher SES, PPD symptoms, and room-sharing may be explained by other factors, such as employment status. Mothers with higher education are more likely to be working (Knop, 2019). Working mothers with more education are also more likely to have private insurance and not receive WIC food assistance during their pregnancy, characteristic of the higher SES subgroup. In dual-income households, women often take on more infant caretaking at night than the father (Burgard, 2011). Additionally, when parents room-share with their infant, paternal nighttime interactions with the infant tend to decrease (Volkovich et al., 2018). These effects would be seen only in women who are married or partnered, and partnered women may have expectations towards a partner’s role in infant caretaking. Less social support during the postpartum period is also correlated with PPD (Vaezi et al., 2019), suggesting that women who are partnered, working, and room-sharing may not perceive sufficient social support from their partner, contributing to PPD symptoms.

Another explanation considers women’s lived experiences from different socioeconomic and cultural backgrounds. A smaller study found that women who co-slept (bed-sharing and/or room-sharing) for more than six months reported more social criticism regarding their parenting decisions than women who did not co-sleep (Shimizu & Teti, 2018). This conflicting pressure to adhere to sleep guidelines and the Western norm of sleeping in separate rooms can certainly contribute to maternal stress. For instance, for many women in Western countries, there is overwhelming advice to encourage infants to sleep through the night, which may be difficult while room-sharing, while sleeping through the night may not be expected in other cultures (Barry, 2021). Room sharing is much more common in non-western groups and women of lower SES (Teti et al., 2022), and it is possible women of higher SES face more or simply different societal pressures regarding infant sleeping arrangements, leading their infant’s sleeping arrangements to influence their mental health. In many Eastern cultures, it is common for grandparents and family to assist greatly in childrearing, which may make room-sharing at nighttime feel less burdensome due to the difference in social support during the postpartum period (Teti et al., 2022; Barry, 2021). Women of lower SES may also be more accustomed to room-sharing during other times of life and may consider room-sharing as less of a change than women who are less accustomed to room-sharing. These women are also reported to face more significant adverse life events than mothers of higher SES (Mersky et al., 2021) and thus may not consider room-sharing to be a significant stressor compared to other stressors they face.

Though this work finds that room-sharing is associated with PPD symptoms, it is imperative to consider the patient’s unique circumstances when determining recommendations to give families regarding room-sharing, as there are certainly many benefits to room-sharing for both mother and infant. Among those is breastfeeding practice, which is theorized to increase when room-sharing due to proximity to the infant at night. A systematic review found that room-sharing may increase the duration of breastfeeding, though the evidence is considered low-quality, likely due to most research focusing on bed-sharing (Ng et al., 2019). Breastfeeding is protective against PPD and has been found to lower the risk of PPD in multiple works (Xia et al., 2022). It is also important to consider that room-sharing does significantly reduce the rate of SIDS, and while there is a modest association between room-sharing and PPD symptoms, this may not always outweigh the two-fold increase in SIDS risk. Additionally, the potential association between SIDS and PPD should be considered when determining whether to room-share. The association between room-sharing and PPD may be modest for the general population, but for individual families, it may be significant. Shared decision-making is important when determining recommendations for each family’s sleeping arrangements, as the costs and benefits for room-sharing may differ greatly based on the family’s cultural norms, background, and individual risk factors.

This study has several limitations. First, we used maternal self-report to define PPD symptoms. These symptoms may have been under or overreported. Second, the cross-sectional nature of the study limits our ability to draw conclusions regarding the temporal nature of associations observed and that reverse causation could have confounded the reported results. Third, the question on infant-mother room-sharing status was limited to the past two weeks, and data on the exact infant age of room-sharing practices and frequency of infant-mother room-sharing within the last two weeks was not captured in the PRAMS. This meant that women who only room-shared for a few days and those who room-shared every day were considered room-sharing in the study. Fourth, although only half of the participants indicated that they were breastfeeding at the time of the survey, data on breastfeeding practices stratified by infant-mother room-sharing status was not captured. Controlling whether a mother ever breastfed may not capture the ongoing breastfeeding experience that could interact with infant-mother room-sharing and potentially influence PPD symptoms. Fifth, women who did not have responses for infant sleeping location, PPD symptoms, or any covariates used in the statistical analyses were omitted from the study. This subset of women may impact the overall results, as nonresponse may be due to current PPD symptoms. Finally, the PRAMS dataset uses questions from the PHQ-2 to assess PPD symptoms, but with slightly different answer choices, making it challenging to compare its sensitivity and specificity to other tools. This highlights the need for studies that compare the PRAMS PPD screening to other well-known tools. Despite this, the questions are commonly used in the PHQ-2 and PHQ-9, and the PRAMS dataset provides a unique opportunity to study both variables of interest using a large sample focused on maternal and infant health. The strengths of our study include its population-based nature, large national sample of racially and ethnically diverse US women, and the ability to adjust to numerous potential confounders.

PPD can have long-lasting impacts on mothers and their families. It is considered a public health issue, and thus it is pertinent to better understand factors influencing PPD. Since the AAP updated their infant safe-sleep guidelines to recommend infants sleep in the same room but in a separate bed as their parents until at least 6 months old, assessing the implication of this on mothers’ mental health is important. The mechanism behind the influence of room-sharing on PPD is not well understood, and further research is needed in this area. Additional research using the DSM-V criteria for PPD is needed to confirm or refute the current findings. Future studies should also explore why the observed association was limited to women of higher SES. Healthcare providers should consider assessing perceived sleep quality and social support among postpartum patients experiencing PPD symptoms. If sleeping arrangements contribute to these risk factors, discussing altering sleeping arrangements through shared decision-making with the family may be beneficial.

## Conclusions

This population-based study describes the association between infant-mother room-sharing and PPD symptoms. The prevalence of PPD symptoms is higher in women who practice room-sharing compared to those who do not. This association was observed only in women of higher SES. The mechanism by which room-sharing is related to PPD is still unknown. Plausible mechanisms include perceived sleep quality, perceived social support, employment, and mother’s lived experiences. By better understanding such risk factors for PPD, public health organizations can align their recommendations and improve maternal mental health.

## Data Availability

The datasets used during the current study are available from the CDC PRAMS website https://www.cdc.gov/prams/prams-data/researchers.htm. Permission and registration are required by the CDC PRAMS to access data.
